# The Spiral Model of Evolution: Stable Life Forms of Organisms and Unstable Life Forms of Cancers

**DOI:** 10.3390/ijms25179163

**Published:** 2024-08-23

**Authors:** Andrzej Kasperski, Henry H. Heng

**Affiliations:** 1 Department of Biotechnology, Laboratory of Bioinformatics and Control of Bioprocesses, Institute of Biological Sciences, University of Zielona Góra, Szafrana 1, 65-516 Zielona Góra, Poland; 2 Center for Molecular Medicine and Genetics, Department of Pathology, Wayne State University School of Medicine, Detroit, MI 48201, USA

**Keywords:** chromosome instability, evolution innovation and conservation, information management, spiral model of evolution, treatment-induced resistance, two-phased evolution, unstable supersystems

## Abstract

If one must prioritize among the vast array of contributing factors to cancer evolution, environmental-stress-mediated chromosome instability (CIN) should easily surpass individual gene mutations. CIN leads to the emergence of genomically unstable life forms, enabling them to grow dominantly within the stable life form of the host. In contrast, stochastic gene mutations play a role in aiding the growth of the cancer population, with their importance depending on the initial emergence of the new system. Furthermore, many specific gene mutations among the many available can perform this function, decreasing the clinical value of any specific gene mutation. Since these unstable life forms can respond to treatment differently than stable ones, cancer often escapes from drug treatment by forming new systems, which leads to problems during the treatment for patients. To understand how diverse factors impact CIN-mediated macroevolution and genome integrity–ensured microevolution, the concept of two-phased cancer evolution is used to reconcile some major characteristics of cancer, such as bioenergetic, unicellular, and multicellular evolution. Specifically, the spiral of life function model is proposed, which integrates major historical evolutionary innovations and conservation with information management. Unlike normal organismal evolution in the microevolutionary phase, where a given species occupies a specific location within the spiral, cancer populations are highly heterogenous at multiple levels, including epigenetic levels. Individual cells occupy different levels and positions within the spiral, leading to supersystems of mixed cellular populations that exhibit both macro and microevolution. This analysis, utilizing karyotype to define the genetic networks of the cellular system and CIN to determine the instability of the system, as well as considering gene mutation and epigenetics as modifiers of the system for information amplification and usage, explores the high evolutionary potential of cancer. It provides a new, unified understanding of cancer as a supersystem, encouraging efforts to leverage the dynamics of CIN to develop improved treatment options. Moreover, it offers a historically contingent model for organismal evolution that reconciles the roles of both evolutionary innovation and conservation through macroevolution and microevolution, respectively.

## 1. Introduction

Cancer, as an aggressive and complex evolving supersystem (Appendix A), continually challenges not only our theoretical frameworks, but also our technical platforms, experimental designs, data interpretation, and clinical expectations—even after over 50 years of the war on cancer, which has led to millions of publications and countless claimed breakthroughs [1]. Due to the contribution of numerous factors to cancer evolution [2] and the chaotic nature of cancer evolution, characterized by high unpredictability [3,4,5], much of the current cancer research is based on predefined linear models and experimental designs. This approach yields valuable data for local or partial characterizations rather than comprehensive system-level insights. However, this approach overlooks the chaotic evolutionary process, which is not a gradual stepwise process, but rather a two-phased chaotic process (Appendix A), which is pivotal for understanding cancer and especially for effective medical intervention.

One typical example is research based on cancer gene mutations. Throughout history, the cancer gene hypothesis has continuously evolved, with limited clinical value for the majority of patients (Appendix A). It began with initial oncogenes and tumor suppressor genes, expanded to include cell death genes and developmental genes, and has further progressed to encompass genes associated with metabolic, immunological, and microenvironmental profiles—each exhibiting vast and intricate combinations. Given the extensive interconnections among the majority of these genes, gene-based research is projected to endure for another 50 years, if not more, albeit shaped by different prevailing hallmarks. Interestingly, the gene mutation theory of cancer, which forms the conceptual basis of current molecular cancer research, has been challenged by both the findings of cancer genome projects and increased appreciation of alternative concepts [6,7,8,9,10,11,12,13,14,15,16,17,18,19,20,21,22,23,24,25,26,27,28,29,30]. The central challenge revolves around establishing a unifying framework capable of harmonizing and advancing this diverse field. Such a framework should not only provide a coherent interpretation of individual case studies but, more importantly, offer valuable guidance for clinical implications.

The most promising strategy involves applying evolutionary concepts and platforms to comprehend and address cancer. Within this framework, a range of genomic and non-genomic factors can be unified as triggers that promote genome or chromosome instability (CIN), either by amplifying the cancer system dynamics or reducing environmental and other evolutionary constraints. Some of the resulting unstable genomes may have the potential to undergo macroevolutionary transformation and genome stabilization, followed by microevolution through clonal expansion and clonal selection along with optional gene mutations as an associated phenomenon. This pattern is often termed the two-phased cancer evolution, where genome chaos plays a critical role in driving the phase transition [31,32].

Both cancer evolution and organismal evolution (phylogenesis) can be described as a cyclical series of two-phased evolution [3,4,26,31,33,34,35,36]. Considering each cancer as a mixed system or a supersystem (Appendix A) that consists of populations of transformed cells that can belong to different systems, the occurrence of macroevolution during cancer formation is responsible for the creation of new systems [32]. The creation of new systems during macroevolution, followed by the growth via clonal expansion within a gene-based microevolution, can result in the emergence of a new supersystem that consists of transformed cells, as well as heterogeneous populations with altered genomes (additionally, see Section 5.2). In the case of cancer, the continuous generation of new systems and the resulting emergence of further new supersystems are crucial for processes such as transformation, metastasis, and drug resistance. It should be noted that in organismal evolution, the emergence of new systems plays a vital role in major evolutionary innovations, including speciation [37]. Interestingly, organic codes play an important role in these innovations, and specifically, the accumulation of various organic codes contributes to increased complexity and diversity [32,38], as most major evolutionary innovations are achieved by the introduction of new organic codes (Appendix A).

Significant efforts have been made to integrate cellular states, genome integrity, organic codes, environmental context, and speciation, including cancer formation, using information and genome-based evolutionary frameworks [31,39,40,41,42]. One specific concept that has been proposed is information management, which encompasses the creation of information at both the part and the system level, its preservation, including storage and transmission, modification, and usage [31,32]. These four components explain the causes and consequences of macro and microevolutionary transitions throughout the natural history of evolution on Earth, encompassing the complexity and diversity mediated by information flow. These transitions include the emergence of the first prokaryotic cell, the first eukaryote, the first multicellular life, the first animal body plan, and, eventually, us. In essence, these historically contingent evolutionary innovations occur through a series of macro and microevolutionary phase transitions, where information is created and preserved within organic codes [32,38]. This model also explains how biological systems build up complexity and diversity by recruiting additional innovations through the creation and preservation of organic codes.

To further illustrate the continuous relationship between major evolutionary innovations and their preservation and copying or amplification through the growth of cell populations, each of which can be linked to various molecular mechanisms, this article proposes the spiral of life function model (Appendix A). This model is designed to provide better visualization of these complex evolutionary phenomena. Additionally, cancer can exhibit seemingly reversed phenotypes during its evolution, such as switching bioenergetic patterns and biased gene usage towards unicellular rather than multicellular profiles [43]. The proposed spiral of life function model can effectively convey the message that by interrupting higher-level constraints (e.g., the genome level), lower-level actions (the gene or epigenetic levels), many of which dominated in earlier evolutionary history, can gain dominance in the altered system. This analysis emphasizes the importance of system information creation and preservation during the cycles of two-phased evolution, which clearly explains the interaction between various molecular mechanisms, genomic instability including CIN, and information management (Appendix A).

Although cancer evolution is no longer a novel concept, since it was introduced in the 1960s [44,45,46], understanding how evolution operates in cancer and how to apply evolutionary principles to guide research remain ongoing challenges. Although not apparent to many, one of the biggest challenges lies in the limited understanding of the genomic basis of cancer, stemming from the confusion surrounding the mechanisms of macro and microevolution. Despite the genome architecture theory (Appendix A) offering an explanation for synthesizing two-phased cancer evolution—comprising stress-induced genome chaos and stochastic clonal expansion—and the information management required to understand cancer at a systemic level, mainstream researchers, unfortunately, continue to prioritize specific molecular mechanisms defined by gene mutations. This focus on gene mutations overlooks the broader insights provided by the theory. For instance, researchers often favor genes associated with metabolic pathways or the transition from unicellular to multicellular states, as both transitions are linked with cancer. However, the association of genes with cancer needs to be reexamined using the two-phased cancer evolutionary model, and the spiral of life function model proves to be highly instrumental in defining this point (Appendix A). Specifically, the spiral model can illustrate the relationship between key cellular features (metabolic type and multicellular state) and macroevolution-mediated organic codes, along with the diverse gene mutations for microevolution [31,32]. In other words, the spiral model, integrating a series of two-phased evolutionary events, within the context of historically contingent coded innovations, can explain chaotic processes, information creation and preservation, genotype and phenotype emergence, and increased complexity.

This article begins with a brief review of evolutionary studies of cancer, focusing on two-phased cancer evolution and emphasizing the significance of information management in creating new cellular systems with new organic codes [3,31,38]. Following this, we introduce the spiral model of key evolutionary innovations or functionalities, which encompass new bioenergetic, unicellular, and multicellular phenotypes. This model is further discussed in the context of cycles of two-phased evolution, in which processes such as information self-creation, preservation, modification, and usage play a pivotal role in the emergence of the new cellular supersystem of cancer. Cancer, as a highly unstable supersystem, lacks strong information preservation mechanisms, unlike most organisms. This instability leads to continuous changes, both at the system level (karyotype) and at the parts level (genes), all within a stable host maintained by sex and developmental processes. As a result, cancer presents an extremely challenging target for treatment and understanding. As the extremely unstable supersystem of cancer literally forms its own robust ecosystems (Appendix A), it is likely to have advantages over the stable evolution of organisms, especially under high levels of stress. Last, but not least, the significance of genome instability or CIN is also discussed, along with its clinical implications.

## 2. The Key Features of Spiral Model of Accumulated Life Functions

**Figure 1:**
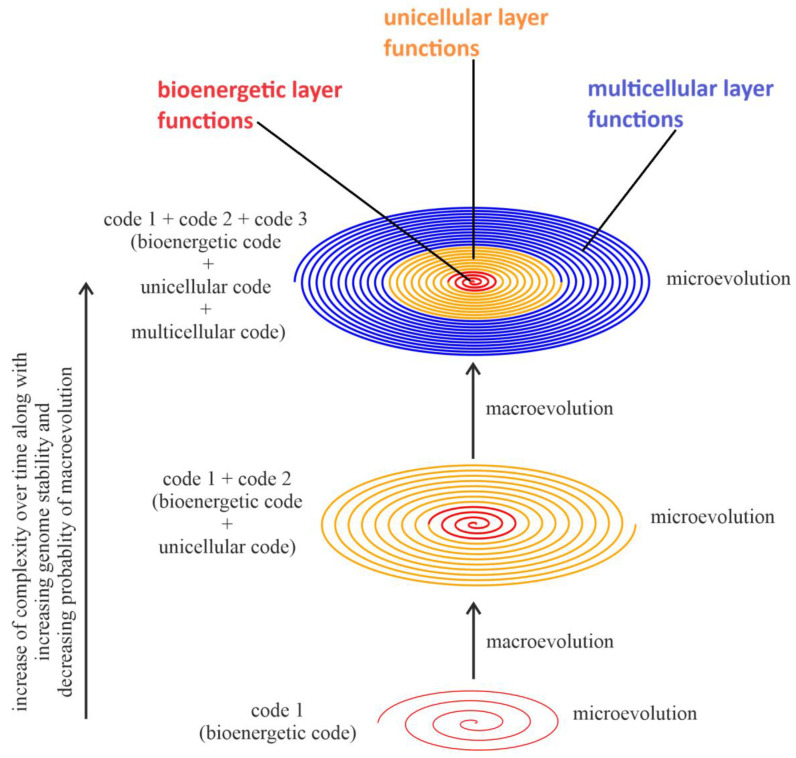
Schematic view of emergence and evolution of unicellular and multicellular organisms. The figure highlights the ways in which new organic codes can be added up to the spiral of life functions, along with historically linking different organic codes. The spiral of life functions of multicellular organisms consists of three main layers: the bioenergetic layer, the unicellular layer, and the multicellular layer. The red part of the spiral contains bioenergetic functionalities (i.e., sets of functions that realize functionalities related to bioenergetics). The orange part of the spiral contains unicellular functionalities (i.e., the set of functions that realize functionalities related to the lives of unicellular organisms). The blue part of the spiral contains multicellular functionalities (i.e., sets of functions that realize functionalities related to multicellularity). The higher-level (external) functionalities of the spiral extend and control the activities of the lower-level (internal) functionalities. The continuous feature of the spiral model not only effectively connects various evolutionary innovations to evolutionary history, but also bridges macroevolution and microevolution.

During evolution, a simple life form can evolve to become more complex, as reflected by the accumulation of phenotypic complexity. This increased complexity is associated with the accumulation of different organic codes [32,38,47,48,49,50,51]. In accordance with the proposed concept of the spiral of life functions, complexity during evolution increases by formation (mainly during macroevolution) and adding to the spiral organic codes of new functions that realize additional (larger) tasks with increased complexity or diversity (i.e., functionalities). Numerous types of functionalities, coded by organic codes, developed across the history of the evolution of organisms on Earth. However, in order to focus on key features related to cancer, this article concentrates on three of them. The concept of the spiral organizes the organic codes of cell functionalities in multicellular organisms into three main functional layers: the bioenergetic, unicellular, and multicellular layers (Figure 1, Appendix A). In this model, unicellular layer functionalities are constrained (controlled) by multicellular layer functionalities, while bioenergetic layer functionalities are constrained by both unicellular and multicellular layer functionalities. This is consistent with the previously published multilayer sandwich model of selection and self-organization, in which higher levels exert greater influence [3,26], as well as the layered model of cellular functionality evolution [39]. It should be noted that the spiral of life functions of unicellular organisms consist of only two main layers, i.e., the bioenergetic layer and the unicellular layer.

In general, in the light of the concept of the spiral of life functions, the evolution of normal cells involves the following:

(a) The formation of new functionalities coupling with new organic codes via macroevolution;
(b) The extension and modification of existing spiral functionalities via microevolution;
(c) The improvement of constraint (control) over the already existing spiral functionalities and functions, i.e., improvement of control over intra-spiral functionalities/functions, which is schematically presented in this article by an increase in the squeeze of the spiral of life functions;
(d) The improvement of genome stability by improving control over the functionalities of the bioenergetic layer, resulting in a decreased probability of the occurrence of macroevolution.

Macroevolutions occur rapidly at the points of major innovations, represented by color changes in the spiral of life functions—the transition from red to orange, and from orange to blue. These color shifts signify the punctuated formation of new systems with increased complexity. In contrast, microevolution lasts for much longer periods, symbolized by numerous spiral turns. During microevolution, the functionalities are modified by the appearance of small changes of a random character, which, along with natural selection, result in a better adaptation of the organism’s cells to the environment. Despite their extended duration and modifications, most microevolutionary changes fail to accumulate over extended periods due to the constantly changing environments, which often oscillate back and forth in the long term. Therefore, the specific mechanisms of microevolution are essential for maintaining the survival of the population in the short run, but they make limited contributions to macroevolution. This recognition has been discussed in other publications [3,31,32,47].

The functionalities located in spiral turns cover the entire evolutionary organism history (with both evolutionary phases, including the pro-cell and the highest cultural organic codes). The functionalities stored in spiral turns can be responsible, among aspects, for energy mechanisms, cell differentiation, and body plans. In general, the functionalities located in the external spiral turns extend and control the functionalities located in the internal spiral turns. This indicates that the evolution of multicellular organism development results in an increase in control over unicellular layer functionalities by multicellular layer functionalities, and also an increase in the control over bioenergetic layer functionalities by unicellular layer functionalities and multicellular layer functionalities. Additionally, in multicellular organisms, the organic codes of all three types of functionalities are preserved by the karyotype code, as it serves as the inheritance platform. This means that karyotype coding, as a concept for storing functionalities associated with chromosomes [3,26,47], acts as a constraining factor for the stability of system-level inheritance. Moreover, the mechanism of chromosome stability and karyotype coding plays a crucial role in information management, including the preservation of information at all the layers and the constraints of all the layers of the spiral of life functions. A decrease in the highest layer’s constraint leads to a decrease in control over the functionalities of the lower layers, resulting in an increase in the dynamics of the functionalities of the lower layers. An increase in the highest layer’s constraint leads to an increase in control over the functionalities of the lower layers, resulting in a decrease in the dynamics of the functionalities of the lower layers. For example, a stable genome can suppress gene mutations [52]. This indicates that an increase in the activity of multicellular layer functionalities causes a decrease in the activity of unicellular layer functionalities, i.e., the correlation between the activities of multicellular layer functionalities and unicellular layer functionalities is negative during normal multicellular organism development. This conclusion can be supported by the work of Trigos, presenting that during the evolution of multicellular organisms, an increase in the number of transcriptomes of multicellular genes causes a decrease in the number of transcriptomes of unicellular genes [43,53].

## 3. The Spiral Model of Cellular Evolution

It is intriguing to explore what constitutes the fundamental difference between cancer and organismal evolution. Traditionally, the pattern of cancer evolution is thought to occur, much like organismal evolution, through the accumulation of small changes over time, with the key distinction that cancer evolution can manifest itself clearly within a single generation of the host, whereas most organismal evolution typically requires significantly longer periods [3,4]. Moreover, it is the normal cells of multicellular organisms undergoing cancerous transformation that constitutes the beginning of this serious disease [54]. Unexpectedly, this difference arises from the contrast in genome stability. In most organisms that can host cancer, the genomes remain rather stable, ensured by the function of sexual reproduction [26,55,56]. In contrast, in the evolution of somatic cells, cell populations do not face strong genome-level constraints, allowing cellular macroevolution to occur much more easily. Further studies have concluded that somatic evolution and organismal evolution actually follow the same two-phased cancer evolutionary patterns. They all involve the introduction of new organic codes and genome-alteration-mediated macroevolution, followed by microevolution. The most innovative features of cancer can be illustrated in the spiral of life function model, incorporating different organic codes (i.e., codes that code different functionalities), including bioenergetics, unicellular formation, and multicellular formation.

### 3.1. The Spiral Model Can Integrate Key Life Functions

One of the fundamental conditions of life is producing energy. For this reason, at the beginning of evolution, simple functions, coded by simple molecular-level mechanisms, associated with bioenergetics (i.e., bioenergetic functions) had to be developed. Subsequently, early in evolutionary history, a transition from the molecular to the cellular level may have occurred naturally [57]. This is consistent with the concept that Darwinian evolution could have started at the molecular level and then progressed to the level with a membrane boundary (i.e., protocells) [57]. Bioenergetic functions were vital for maintaining the lives of these first primitive unicellular organisms and to reproduce them. Note that the creation of a first life system differs from copying a newly formed life system to form a population. The creation of a system is a macroevolutionary process, distinct from the assumption that small changes lead to macroevolution. It involves the drastic emergence of system-level changes, often associated with the formation of a new genome. The emergence of the first life forms can unequivocally be classified as macroevolution. In contrast, copying this system via biological mechanisms belongs to microevolution as the system is preserved, and only small changes, such as gene-level alterations, are involved. In this way, during the organismal evolution of normal organisms, a hierarchical organization of cellular functionalities can be formed through two-phased evolution. Additional key functionalities can be added on top of the previous ones, leading to increased complexity, which is ensured by a chain of multiple layers of codes. In this article, the process of the creation of a hierarchical organization of functionalities by adding up different organic codes during evolutionary cycles, in which higher-level functionalities extend and control lower-level functionalities, is schematically presented in the form of the development of the spiral of life functions (Figure 1). It should be noted that this concept is also in accordance with the layered model of the evolution of cellular functionalities and, in general, is in line with phylogenetic memory phenomenon [39,58,59].

In the light of the concept of the spiral of life functions, the occurrence of macroevolution results in a new system, with huge potential for small modification within the existence of the genome. An increase in the number of turns of the spiral represents microevolution within the boundary of macroevolution. During the microevolution of normal organisms, populations of organisms of the same type (i.e., organisms belonging to the same system created during macroevolution) are grown, along with small adaptive modifications and the preservation of the system’s accumulated information. In this context, the system strives, over time, to achieve population survival plus microevolution-selected complexity through the creation, modification, and accumulation of parts-level information, as well as the preservation of old information, which allows for effective the self-copying of the accumulated information that defines this system. Subsequently, the rapid creation of system information, along with the gradual modification of parts and the integration of previous information, leads to the emergence of increasingly complex organisms with enhanced functionalities. According to the concept of the spiral of life functions, during the evolution of the normal cells of multicellular organisms, increasingly effective control over unicellular layer functionalities and bioenergetic functionalities is established. This highlights that when a new organic code is created through macroevolution, it must be preserved, and the constraint force ensures microevolution. An increase in the number of multicellular functionalities during the evolution of multicellular organisms is related to an increase in the control over intra-spiral functionalities, including an increase in the control over unicellular and bioenergetic functionalities. In accordance with unified cell bioenergetics, this increase in control, especially over bioenergetic functionalities, results in a decrease in the risk of cell overenergization, resulting in genome destabilization [41]. The increase in control over intra-spiral functionalities, the related decrease in the risk of genome destabilization, and the resulting decrease in the probability of the occurrence of macroevolution indicate that during normal organism evolution, genome stability increases. This could be one of the reasons why today, multicellular organisms seem stable of being trapped in their genome attractors [40,60,61]. This conclusion is consistent with the current research interest in organisms as attractors in phase space [62]. Trapping in the separated genome attractors allows for the stabilization of the configuration of features characteristic of given organisms through microevolution [61].

### 3.2. Cell Bioenergetic Problems as a Cause among Many of Cancer Transformation

In accordance with unified cell bioenergetics, bioenergetic problems related to cell overenergization can cause the activation of the Crabtree effect and disturbances in current cell fate, leading to an increase in the probability of changing the cell fate to cancerous cell fate, along with activation of the Warburg effect to reduce the overenergization of mitochondria [39,40,41,63,64]. This switch to cancerous cell fate (i.e., cancer transformation) can occur without any mutations, as a result of huge disturbances in the operation of the functionalities of the multicellular layer, leading to an uncontrolled loss of control over unicellular layer functionalities and activation, among others, with the Warburg effect as a result [39,65,66,67]. The switch to cancerous cell fate can also occur with mutations as an associated phenomenon [39,41,63]. It is known that cancer transformation arises from a combination of environmental and genetic factors [68]. Long-term smoking, alcohol abuse, some infectious diseases, poor eating habits, and a sedentary lifestyle are all risk factors [3,69]. These risk factors can affect the environment and change it to a procancerous environment (i.e., an environment that is more conducive to cancerous transformation). The switch to a cancerous cell fate can occur when in given (i.e., existing at a given moment) intracellular and extracellular conditions, the mutations (i.e., sets of mutations) cause huge disturbances in the operation of multicellular layer functionalities, leading to an uncontrolled loss of control over unicellular layer functionalities [39]. It should be noted that the same set of mutations in different intracellular and/or extracellular conditions may not cause huge disturbances in the operation of multicellular layer functionalities and, for this reason, will not cause cancerous transformation. It can be easily concluded that exactly the same set of mutations can be found in cancerous cells and in healthy cells, because the condition for the occurrence of huge disturbances in the operation of multicellular layer functionalities leading to uncontrolled loss of control over unicellular layer functionalities is the appearance of the mutations that are needed for the occurrence of these disturbances in given intracellular and extracellular conditions. It can also be concluded that the cell with a given set of mutations can undergo transformation within a very short time after the occurrence of these mutations, and that it can also “wait” for a very long time, for example, for several years, for the appropriate intracellular and extracellular conditions for the occurrence of huge disturbances in the operation of multicellular layer functionalities, which increase the probability of transformation. A special form of cancerous transformation is transformation that occurs without any mutations, when given intracellular and extracellular conditions are sufficiently bad to cause huge disturbances in the operation of multicellular layer functionalities to occur. In view of unified cell bioenergetics, cell overenergization (as one of the factors worsening living conditions) significantly increases the risk of cancer transformation.

### 3.3. A Phenomenon of Cancer Transformation as a Result of Disturbances in the Spiral of Life Functions

The spiral of life functions not only allows for general descriptions and predictions regarding the evolutionary process of normal organisms, but can also apply to cancer research. In opposition to the development of cells of normal multicellular organisms, during cancer progression, the uncontrolled deterioration in (or the uncontrolled loss of) control over the functionalities of unicellular layer occurs. This phenomenon occurs as a result of huge disturbances in multicellular layer functionalities, resulting in a decrease in the activity of multicellular layer functionalities. For this reason, during cancer development, unicellular layer functionalities, which are normally controlled by multicellular layer functionalities, can attain uncontrolled activities. This indicates that a decrease in the activity of multicellular layer functionalities causes an increase in the activity of unicellular layer functionalities, i.e., the correlation between the activities of multicellular layer functionalities and those of unicellular layer functionalities is also negative during cancer development (additionally see the Section 2). This conclusion can be supported by the work of Trigos, which suggests that a decrease in the number of transcriptomes of multicellular genes causes an increase in the number of transcriptomes of unicellular genes [43,53]. It should be noted that an increase in the activity of unicellular layer functionalities related to cancer progression is associated with an increased risk of cell death, which can be also supported by the work of Trigos [53].

The activation of unicellular genes in cancer cells could also be explained by their strategy of differentiating from normal tissue in order to gain a competitive advantage, rather than reverting back to a unicellular state. Cancer is a tissue-level-based system, not a single-cell one. It is important to note that the genomes of developed multicellular organisms cannot revert back to a unicellular state due to the extraordinary complexity of developed multicellular organisms’ genomes. Moreover, during cancer progression, massive changes (due to macroevolution) to the cancer genome alter the original genome, i.e., the genome of the cell before transformation (additionally, see the Section 5.5). Cancer cells retain their identity as cells with multicellular tissue organization, albeit differing from normal tissue. This identity remains distinct from those of unicellular organisms, like amoebas [31,32,70].

Based on the spiral model (Appendix A), the key is to remove normal system constraints by altering the karyotypes, and the three levels of organic codes will be changed accordingly. Specifically, under normal conditions, karyotypes remain stable, governed by constraints imposed by both the tissue and the overarching system. This intricate interplay between system-level and part-level inheritance guides the functioning of the organism. However, when cells face stressful conditions, cancer, considered as a system, adapts. The emergence of altered karyotypes gives rise to new organic codes, breaking free from previous constraints and adopting novel functions—divergence being the key strategy for cancer cells to thrive. While the majority of these newly formed systems are swiftly eliminated by the host’s defense mechanisms, outliers that withstand the macroevolutionary test can become stabilized. They create a foundation for microevolution, blossoming into a population of cancer cells. When profiling these late stages, the gene mutations are obvious. However, in the crucial phase of early macroevolution to create the first cancer cells, it is not these genes that play the key role. In essence, the disruption of constraints, including, but not limited to metabolic and multicellular states, leads to the dominance of these new karyotype-defined systems—a narrative that unravels how cancer arises, forging a path marked by evolutionary innovations. Fortunately, their genetic legacy remains unable to endure and propagate to new hosts, at least for now.

### 3.4. Chromosome Instability as a Common Cause of Cancer Transformation

In accordance with the karyotype coding concept, functionalities are stored in chromosome sets, or the topological configuration of genes on chromosomes. For this reason, CIN can cause very radical changes in the whole set of functionalities, in addition to linking with many molecular pathways of cancer [71,72,73,74,75,76]. This is also illustrated by the spiral model. This means that the occurrence of CIN can result in the physical restructuring of the entirety of the spiral of life functions (i.e., the occurrence of CIN and the resulting genome chaos and massive genome remodeling can lead to changes in the number of turns of the spiral of life functions, huge relocations of the functionalities inside the structure of the spiral, huge modifications of the functionalities, the deactivation of existing functionalities, and also the creation of new functionalities). The occurrence of CIN can cause changes in the multicellular layer functionalities that normally control unicellular layer functionalities. This can lead to the uncontrolled loss of control over unicellular layer functionalities, which can be immediate and can involve a very large number of ancestral functionalities. Moreover, the occurrence of CIN is related to the huge modification of functionalities originally stored in the spiral, including unicellular layer functionalities. For this reason, a huge cohort of altered functionalities of the unicellular layer can dominate the life of a transformed cell as a result of the occurrence of CIN. These phenomena allow cancer cells to choose different metabolic pathways, which are often altered or are unavailable before cancer transformation. This is also why nearly all transformed cells display altered karyotypes, and it is why the CIN-mediated macroevolution observed in most cancers allows the breaking of the host’s karyotype and generate new ones with phenotypes that are better adapted to the microenvironment and suited to winning at evolutionary selection. The association of gene mutations mainly occurs in the microevolutionary phase to grow the population [31]. Furthermore, the overall stability of the host can impact the dynamics of the spiral, leading to changes in the speed (either speeding up or slowing down) of evolution. On one hand, the aging of the host can reduce system constraints on cancer, increasing cancer incidence. On the other hand, aging-related reduced function requires genome-level changes for functional compensation, which also increases opportunities for cancer evolution as a by-product of evolutionary adaptation. It is known that many more genome and gene alterations are detected in aging tissues.

Clearly, chromosome or genome stability is vital for an organism to maintain its identity and reduce the likelihood of cancer evolution mediated by cellular variability. To overcome evolutionary constraints imposed by the host, variable cells must rely on the mechanism of CIN to create new cellular systems and compete with host cells.

### 3.5. The Cancer Supersystems Represent New Cellular Ecosystems

The spiral model also explains why cancer cells can be highly aggressive, especially following various harsh medical treatments. While each normal species occupies a fixed region of the spiral, a cancer cell population with diverse epigenetic landscapes can simultaneously occupy many locations on the spiral. This ensures a highly dynamic potential for both macro and microevolution. The formation of supersystems has obvious advantages, as they literally create different ecosystems within cancer microenvironments. In these ecosystems, different cell populations often represent distinct cellular species, mixed with varying sizes of clones, engaging in both macro and microevolution. Furthermore, the high rate of cell death is coupled with information self-creation through various mechanisms of genome chaos, including chromothripsis, massive translocation, micronuclei clusters, chromosomal fragmentations, and polyploid giant cancer cells (PGCCs) [32,70,72,77,78,79,80,81,82,83]. Within these supersystems, closely connected cells are constantly involved in DNA transfer, cell fusion, and atypical cell division, increasing the opportunities for forming more fitted clones with aggressiveness and drug-resistant features [84,85,86].

## 4. Information Management in the Spiral Model

The continuous spiral model suggests that different evolutionary innovations are linked by information flow. Although much research is needed to study the mechanism of information creation and preservation, some mechanisms are well known. For example, there are many ways to alter karyotype coding, such as spontaneous chromosome fusion/fission, hybridization, genome chaos under crisis, transposable elements, and other gene transfer mechanisms [3,31,32]. Similarly, there are many ways to change parts-level information, including gene mutations and epigenetic alterations [38]. Viral infection and organism symbiosis within the host cell that occur in accordance with endosymbiont model can be given only as selected examples. According to the endosymbiont model, mitochondria evolved from a bacterial progenitor via symbiosis within an essentially host cell [87]. Subsequently, almost all of the organic code that was originally in the mitochondria relocated to the nucleus by endosymbiotic gene transfer (EGT), which contributed significantly to nuclear DNA (nDNA) [87,88,89,90,91]. Now, relocated during evolution genes not only encode proteins that are imported to the mitochondria, but are also involved in the addition of new proteins and new functions [87,92]. As a result of gene relocation, most mitochondrial proteins are encoded by nuclear genes [93]. As an example, about 99% (i.e., more than 1500) of mammalian mitochondrial proteins are encoded by the nuclear genome, synthesized as precursors on cytosolic ribosomes, and imported into mitochondria by mitochondrial protein import machinery [93,94]. The processes of importing and sorting of mitochondrial precursor proteins occur across one or both mitochondrial membranes [95]. These processes are catalyzed by the translocases and involve an amazingly versatile set of mechanisms and at least four different pathways [95]. These very complex processes of importing and sorting the proteins encoded by nuclear genes, along with the supporting figures presenting these processes, are depicted, for example, in [93]. In view of the concept of the spiral of life functions, the genes relocated from the mitochondria to the nucleus are injected into the spiral of life functions. Moreover, these injected genes extend functionalities stored in the spiral by forming new functionalities. From this point of view, this type of injection of the new organic code into the spiral of life functions can be called ‘controlled injection’.

Some viral infections are associated with chromosomal anomalies, leading to cancer. Examples include infection by human papillomavirus (HPV) and infection by hepatitis B virus (HVB) [96]. Infection by HPV, which is responsible for 95% of cervical cancers and is also involved in some head and neck cancers, is concluded in the integration of viral sequences into the host genome and is involved, among others, in the generation of CIN [96,97]. Infection by HVB, which is responsible for human hepatocellular carcinoma, is associated with CIN in cancerous and noncancerous liver genomes. HBV–DNA integration is known to be the cause of interchromosomal genomic rearrangements that lead to megabase-size telomeric deletions [96,98]. As a result of the integration of viral sequences with the host genome, today, about 60,000 proviruses in the human genome reveal a history of multiple pandemics [99]. Some of these viral remnants in the human genome still retain the ability to produce viral proteins. Particularly interesting human endogenous retrovirus (HERV) genes are active in tumors [99]. In view of the concept of the spiral of life functions, viral infection causes the injection of external genomic material into the spiral of life functions, causing an elevated risk of CIN, resulting in the destabilization of the functionalities of the multicellular layer, the uncontrolled loss of control over unicellular layer functionalities, and cancer transformation. From this point of view, this cell-uncontrolled, cell-unsafe, and unpredictable injection of the new organic code into the spiral of life functions can be called ‘uncontrolled injection’. Of course, when a system is unstable, controlled injections could also have uncontrolled effects. Viral infections have been linked to increased cancer evolutionary potential, including increased chromosome fragmentations [3], as well as PGCCs [100].

## 5. Selected Aspects of Two-Phased Cancer Evolution

In this section, selected aspects of two-phased cancer evolution are presented and discussed.

### 5.1. The Physical Restructuring of the Existing Genome Can Result from Either the Death or the Emergence of a New Life Form

Cell overenergization results in an increased reactive oxygen species (ROS) level, causing an increase in the speed of the generation of random DNA mutations [41,63]. The accumulation of random DNA mutations can cause the destabilization of the genome, resulting in genome chaos [41,63]. There are nearly unlimited developments that can result in genomic chaos [75,76]. Genomic chaos can lead to significant changes in the genome, resulting in the elimination of many cells. The survivable cells with new genomes must undergo macroevolutionary selection. A small fraction of the selected cells, if their genome stabilizes, will then transition into microevolutionary phases, ultimately giving rise to large populations. This process is therefore linked with massive death and opportunities for the emergence of new life, albeit the change is very low. During this process of genome reorganization, old cell fate must be transformed to a new cell fate (i.e., a new cell fate has to be activated), because massive modifications of the genome cause huge changes in the genetic program written in DNA [31]. This is why evolution can continue like a spiral, despite the information flow through different organisms.

### 5.2. Scenarios of Competition between Clones

The occurrence of macroevolution during cancer progression causes the emergence of a huge number of new systems that are responsible for generating cancerous clouds of cell fates [39,40]. Based on the stepwise evolutionary concept, during competition, the best clones eliminate the weaker ones by natural selection, leading, in this way, to the dynamic and permanent adaptation of a population of clones to the environment and establishing the strongest clones. In reality, the dynamics are much more complicated, as the occurrence of CIN with resulting macroevolution (genome chaos, huge genome remodeling, and the emergence of new systems) causes cancerous clones to compete with each other not only inside thee systems in which they belong, but also with clones that belong to other systems. During this competition in the mixed multiple runs of micro and macroevolution, the states of the “best clones” are constantly changing.

To sum up, macroevolution driven by CIN, along with natural selection, leads to the emergence of new systems and the elimination of weak systems from the supersystem, considering cancer as a whole as a supersystem consisting of transformed cells belonging to different systems. As a result, the longer-term progression of cancer with active macroevolution may lead to the emergence of new systems and the replacement of old systems by stronger systems, which may indicate that not only can new systems emerge during the evolution of cancer, but also that a new supersystem can gradually emerge.

It should be noted that currently, the development of informatics enables the examination of cancer evolution using different computational methods. These methods include, among others, phylogenetic trees, the dot-matrix method, and artificial intelligence methods using data from, for example, cytogenetic and cytogenomic web resources [101,102,103].

### 5.3. Cancerous Microenvironment as a Stimulator of Macroevolution

During cancer evolution, a specific cancerous microenvironment–a new evolving ecosystems—is created and maintained. The cancer microenvironment is a complex and dynamic entity composed of cellular and non-cellular components including secreted factors and the extracellular matrix [104,105]. The cancer microenvironment can induce clonal progression and intratumoral heterogeneity, an increase in multidrug resistance, and the stimulation of metastasis [104,105]. The creation of this microenvironment can be explained using the information presented in the previous sections of this article, as follows: During cancer evolution, many new systems can emerge as a result of macroevolution. Each system contains clones of different mutations, as a result of microevolution. Clones from different systems are evolutionarily very distant, because systems are created as separate processes of macroevolution. This implies that the functionalities of clones belonging to different systems may differ significantly. For this reason, the cell metabolic pathways characteristic of different systems can differ significantly. As a result, the metabolites formed by the cells of different systems can vary greatly. For this reason, the metabolites that leak from cancer cells can be very diverse, different, or new, especially compared to the metabolites that leak from normal cells. These altered, different metabolites are part of the cancerous microenvironment and make up that microenvironment. A significantly changed cancerous microenvironment, compared to the environment of healthy tissue, may additionally stimulate macroevolution, as well as microevolution, causing the stimulation of the growing cancerous microenvironment. This positive feedback occurring during cancer progression can lead to a huge and rapid growth of the cancer microenvironment, resulting in a huge stimulation of cancer evolution.

### 5.4. Cancerous Tissue and Elevated Risk of Metastases Caused by Macroevolution

Normal cells show contact inhibition, whereas cancer cells grow on top of and over each other, disregarding their neighbors [106]. During the process of cancer evolution, which is responsible for creating cancerous tissue, multicellular layer functionalities are disturbed or disrupted, which is the reason why the created tissue is morbid and abnormal. Moreover, damage to multicellular layer functionalities that progresses over time may result in the disintegration of cancerous tissue, leading to an increased risk of metastases [2,107]. From this point of view, the biggest risk of metastases is when macroevolution, caused by genome destabilization followed by genome chaos and huge genome remodeling, occurs during cancer progression. In light of the information presented in this article, the occurrence of metastases means that new systems are activated in a different part of the body of the host organism. The activation of a new system in a different part of body is related to differences in the environment compared to the site of the original occurrence of cancer (i.e., the site from which the cancer spread). Different unstable environments can cause the stimulation of macroevolution and, usually after a longer time, which is needed for the adaptation of the metastatic cell to the environment in a new location, the dynamic progression of cancer is associated with the creation of new systems in the new location. The occurrence of macroevolution during cancer progression indicates not only an increased risk of metastases, but also increased epigenetic plasticity in the adaptation of cancer cells to the environment. The occurrence of macroevolution through genome chaos and massive genome remodeling causes the adaptation of cancer cells to the environment to occur in very genetically different, evolutionarily distant locations in genome space. Additionally, macroevolution, as a very radical process affecting substantial physical changes in the entirety of the spiral of life functions, including the huge destruction of multicellular layer functionalities, can result in the immediate uncontrolled loss of control over large numbers of unicellular layer functionalities, in addition to, as mentioned previously, a significantly elevated risk of metastases. On the other hand, very massive genetic changes causing genome destruction associated with macroevolution can lead to the self-destruction of some systems. It should be noted that, in this case, the probability of the self-destruction of all systems is very small for bigger, evolutionarily advanced cancers containing many systems.

### 5.5. Cancer as a Phenomenon with Many Faces—Cancer Progression Is Not Simply Atavism

Cancer progression is characterized, among other aspects, by the uncontrolled loss of control over unicellular layer functionalities stored during multicellular organism development. Therefore, huge perturbations in multicellular layer functionalities can lead to an uncontrolled loss of control, which is gradual or more or less immediate, depending on the disturbances, over some functionalities stored “at different time depths” (i.e., in different internal turns of the spiral of life functions; see Figure 1), which are characteristic of different ancestral functionalities established during organism evolution. In order to capture the whole picture of the active functionalities occurring during cancer progression, the uncontrolled activity of some ancestral functionalities must also be extended by the possible, albeit disturbed, activity of the multicellular layer’s functionalities. As a result, active functionalities of quite different types (i.e., from different layers) can occur as a result of cancer transformation. Moreover, this cancerous mix of active functionalities is likely to be extended with the possible activation of the functions of viruses that, within the human genome, still retain the ability to produce viral proteins (see the Section 4). A further consideration is the possible occurrence of macroevolution, which through genome chaos and massive genome remodeling, causes enormous physical changes to the entirety of the spiral of life functions, resulting in highly altered genome expression, as well as the creation and activation of new functionalities. As a result, unknown genome expression, which has never existed before, is activated after cancer transformation. This is something like a kind of quasi-atavism, i.e., a new quasi-atavistic system, after which, over time, a supersystem emerges in which a cohort of functionalities, which are usually altered compared to the functionalities of origin, of different types is active. This evolutionary cloning supersystem is constantly modified during two-phased cancer evolution. As a result, this heterogeneous supersystem exhibits great flexibility, unstoppable genomic and nongenomic variability on a wide scale, i.e., from small adaptive changes caused by microevolution to massive changes caused by macroevolution and tendencies to relapse, for example, after radiotherapy and chemotherapy. In short, it is important not to solely rely on a similar phenotype when determining whether the cancer represents reverse evolution back to its ancestor. Cancer is not a unicellular system, but rather a phenomenon of tissue, representing a supersystem with complicated genomes and their expression packages within altered tissues [31,70].

## 6. Conclusions and Future Directions

**Figure 2:**
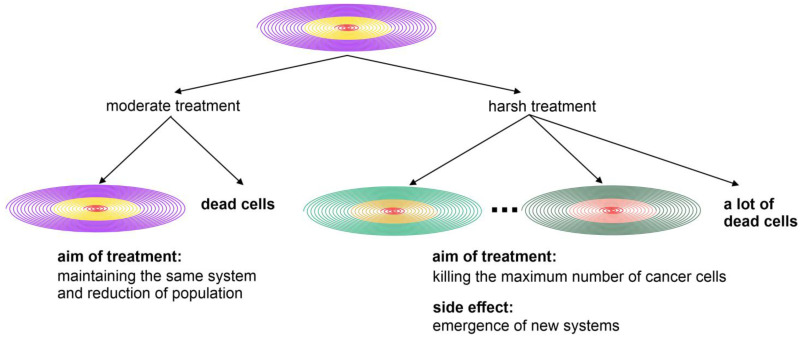
Using the spiral model to explain treatment-induced rapid drug resistance and aggressiveness.

Even though there is a growing consensus that the complexity of cancer can only be fully understood through evolutionary approaches, the question of how to achieve this is still a topic of debate [4,11,12,107,108,109,110]. In recent years, several exciting trends have emerged. First, the concept of two-phased evolution separates macro and microevolution, emphasizing the CIN as a common driver of cancer evolution. Second, the integration of genome chaos and various phenotypes, particularly the PGCCs and micronuclei clusters, has helped to classify human cancer into two major groups with distinctive mechanisms related to the developmental process [13,14,32]. Third, the information management concept has been applied to explain the mechanism of cancer as an information creation supersystem. To synthesize, the spiral model is used to illustrate the necessary integration. The following are some key messages from this article:

(a) A series of two-phased evolutions are linked by the spiral model. With the establishment of major macroevolutionary innovations, more organic codes form, coupled with increased complexity. The preservation and further modification of these codes provides the mechanisms for long and stable microevolution, allowing populations to grow. Therefore, maintaining the integrity of the chromosome sets or genome is important for given species. In contrast, CIN can harm a species, potentially leading to the emergence of conditions such as cancer.
(b) Macroevolution occurs rapidly, as indicated by the sudden color change, while microevolution is a much longer process, represented by the spiral maintaining the same color. The continuity of the spiral among different species is achieved by different organisms, which function as carriers of information. Specifically, for species with sexual reproduction, the sex function acts as a filter to preserve the karyotype coding, which, in turn, also preserves most of the organic codes [47,111,112]. In cancer, the continuity of the spiral is achieved by different populations of cells with different karyotypes. Comparing the phenotype and genotype profiles of cancer with those of the ancestor (normal cell), cancer cells often lose the constraints on normal cell regulation and collaboration, as well as genome integrity.
(c) For all biological entities, whether independent organisms like humans or cellular organisms like cancers, existence is achieved through mechanisms of information creation, preservation, and amplification during the evolutionary process. For example, the success of organismal evolution depends on an individual’s physiological capability to organize and constrain cellular components for the individual’s function, while the success of cancer evolution depends on breaking down these constraints to form new cellular systems that can compete with the host. CIN is a key player in the conflicting relationship between the host and cancer. When the genome is stable, cancer has a lower chance of success, although the host also reduces its capability for cellular adaptation. When CIN is dominant, new cellular systems can more easily overcome constraints and become cancerous. This is why CIN is a common driver of most cancers. The discussion of bioenergetic disturbances fully supports this view, as many molecular mechanisms are linked to CIN. Indeed, the evolutionary mechanism of cancer, which encompasses the collection and combination of all individual molecular mechanisms, is proposed to unify the field.
(d) During the normal evolution of organisms, when new cellular functionalities are added, the dominance of previously existing functionalities is often adjusted. For example, before typical chromosome formation, individual genes in bacteria might have been more dominant. After chromosome formation, the system-level inheritance takes precedence over the part-level inheritance, becoming an organizer to regulate and constrain the functions of individual genes. The increased organic codes also require genome stability to ensure their maintenance. During cancer evolution, CIN breaks outside the spiral of life functions, removing many constraints and allowing the abnormal activity of unicellular genes, for example. The newly formed genomes no longer follow the host’s system control.
(e) The battle between the stable microevolution of hosts and the unstable life form of cancer is the very reason why eradicating cancer using powerful drugs proves to be so challenging. This underscores why a singular focus on eradication may be less effective, as treatment-induced crises can actually promote cancer by increasing the probability of macroevolution and, therefore, the opportunity for these unstable life forms to become more aggressive. Novel approaches to combat cancer should be grounded in the understanding of the two-phased evolution and effective information management, because inappropriate treatment may inadvertently harm the host by creating highly unstable genomes. These pharmaceutically created, more aggressive supersystems consist of a mixture of cells, both clonal and nonclonal, representing different stages of evolution. They become extremely challenging to fight, as each of these genetically distanced systems may require different pharmacological agents to eliminate them. Incorrectly established pharmaceutical agents may extinguish some systems, but can also lead to the stimulation of new cancer populations with altered dynamics of both macroevolution and microevolution.
(f) Information management in cancer and host organisms offers new insights into cancer evolution. Cancers possess numerous abilities to generate new information during their evolution, without the constraints of sexual reproduction. This puts them on different competitive playing fields. Given the inherently chaotic nature of cancer evolution, determining the types of data to collect, how to prioritize research, at which phase, and on what scale of systems are still matters of ultimate importance and necessary to debate. For example, one alternative explanation for the mechanism of adaptive therapy [113,114] could be the reduction in genome chaos events. We anticipate that using moderate treatment to slow down microevolution without triggering massive genome chaos could be a new strategy for managing cancers in the future [3,4,31,84].
(g) Genome chaos needs more mathematical understanding (Appendix A): it is challenging to describe genome chaos using current mathematical tools. Biological chaos, with its unique specificity, is much more complex than most physical systems. However, due to the involvement of developmental, physiological, and evolutionary constraints, its predictability can be greater than in physical systems under certain conditions. The question of how to incorporate these key differences into classical chaos theory requires further research. Nevertheless, an increasing number of papers discussing genome chaos from this perspective are being published [115].
(h) The spiral model helps us to understand the mechanisms of both cancer and organismal evolution. Due to the constraints of the genome landscape, evolutionary selection aims to reduce changes in the host genome. In contrast, for successful cancer evolution, favoring system instability leads to the emergence of new systems, in which the supersystem or cancer ecosystem becomes more aggressive. This information is valuable for designing strategies to combat cancer without triggering genome chaos. An example of this would be paying more attention to treatment-induced drug resistance [72,84]. This approach is likely to explain the success of adaptive therapy as well [113,114], providing new prospects for more effective cancer treatments in the near future. One important mechanism of cancer-drug-treatment-induced resistance can be illustrated using the spiral model. The treatment can focus on either microevolution or macroevolution. Using moderate treatment, the cancer population can be reduced without triggering genome chaos. Even though the initial killing is less remarkable, the long-term benefit can be better. Harsh treatments aimed at eliminating all cancer cells can often trigger genome chaos, leading to short-term massive cell death. However, this aggressive treatment can simultaneously result in the emergence of new cancer systems, via rapid genome reorganization, which are drug-resistant and more aggressive. In a sense, treatment aimed at killing cancer cells can promote the success of cancer macroevolution, leading to drug resistance and aggressiveness. This mechanism can explain why aggressive treatment often has good short-term outcomes, but lacks significant long-term survival benefits (Figure 2).

Figure 2 illustrates the treatment-induced consequences using the spiral model, which explains the relationship between macro and microevolution. This model highlights the evolutionary patterns of different types and scales, including the history of organismal evolution, the dynamics of cancer populations, and various mechanisms within a given species. Color changes represent phase transitions (macroevolution, major evolutionary novelties, different species). In this figure, the related cancer populations are shown with different colors representing different systems. After moderate and aggressive treatments, different evolutionary patterns emerge, with clinical implications. In the moderate targeting group, microevolutionary strategies can reduce cancer cell populations by constraining the cancer. However, while the maximal killing strategy can initially kill many more cancer cells, high-stress conditions can trigger genome chaos, promoting the emergence of new systems that display drug resistance and aggressiveness, followed by rapid microevolution to form dominant populations in the long term [4,5,75,84,116]. This figure suggests the need to reexamine the current maximal killing strategy and identify better evolutionary factors, focusing on long-term survival rather than just short-term gains.

## Data Availability

No new data were created or analyzed in this study. Data sharing is not applicable to this article.

## Appendix A

Definition and Explanation

**Cancer Gene Hypothesis or Gene Mutation Theory of Cancer:** This refers to the hypothesis that cancer results from the accumulation of a few key gene mutations. The traditional view is based on the idea that cancer is a growth disease (out-of-control growth), and that approximately 5–6 gene mutations are needed for a normal cell to become cancerous. Furthermore, it predicts that only a few common gene mutations are responsible for the majority of cancers. Despite decades of extensive studies, this hypothesis is incorrect. Cancer represents a process of new systems emerging, with genome reorganization being the key event. Gene mutations are important only in the second microevolutionary phase, when the initial cancer cells are formed via macroevolution. Many genomic and non-genomic factors, including stochastic gene mutations, can have similar functions in microevolutionary growth, making cancer clinically significant [1,4,8,19,20,21,22,23,24,25,26,108,110,117,118,119,120,121,122,123,124,125,126].

**Genome Architecture Theory:** This refers to a comprehensive framework for understanding the genome’s role in genomics, development, evolution, and disease. This theory shifts the focus from individual genes to the entire genomic system, highlighting the importance of large-scale chromosomal changes, genomic stability, and the dynamic processes driving evolution and disease. It can be summarized by the following concepts [1,9,31,32,127,128,129,130]:

1. **Genome as an Integrated System Rather than a Bag of Genes:** The overall genomic architecture, defined by the karyotype (the complete set of chromosomes), is crucial for maintaining system identity, stability, and functionality.
2. **Karyotype Coding:** Responsible for system inheritance, it organizes gene interactions by defining the genetic network structure. Alterations in karyotype coding play a key role in organismal and cancer evolution.
3. **Genome Chaos:** Represents an effective platform for creating system information, which is essential for macroevolution. Chaotic genome reorganization, confirmed by cancer sequence projects and known by names like chromothripsis, exemplifies this concept [131,132,133].
4. **Two-phased Evolution Unifies Macro- and Microevolution:** Unlike the traditional assumption that macroevolution equals the accumulation of microevolution over time, the mechanisms of macro and microevolution differ. Macroevolution involves genome alterations, while microevolution involves gene mutations. This two-phased evolution model reconciles different molecular mechanisms for both genomics and evolution [109,134,135,136].
5. **Information Management:** Traditional information research has overlooked information creation at the system level. Information preservation is crucial for ensuring self-organization into biological routines. Previously, research focused mainly on genetic code usage. The concept of information management reconciles genome and gene functions, providing a new perspective on evolution as information flow, which also drives evolution [31,32,38].
6. **New Perspective of Inheritance:** The genome defines the bioinformation package, rather than genes, representing a holistic view of genetic information. There is a complex and dynamic relationship between multiple scales of genomic and non-genomic inheritance, with the environment serving as an important information context. All inheritance is fuzzy, coding a spectrum of potential phenotypes rather than a defined phenotype, explaining the gaps between genotype and phenotype. Fuzzy inheritance and environmental dynamics form the basis of biological heterogeneity [4].
7. **New Evolutionary Perspective:** Evolutionary constraint is the major function of microevolution. The function of sex is the preservation of system inheritance [56,112]. Developmental and physiological mechanisms also serve as constraints. Genome constraint and gene dynamics are key to understanding the process of somatic change without germline change under “normal” environments, until new genomes form under crisis. This perspective explains the importance of massive extinction events that drive rapid evolution on Earth and aggressive cancer following maximal treatment [32,84,137,138,139,140].
8. **Redefined Functions of Evolution:** In the macroevolutionary phase, chromosomal instability (CIN), either inherited or induced (often arising from environmental crises, including hybridization and accidental access to foreign genomes), promotes genome reorganization via chaos followed by macroevolution. Once new systems or species are formed and survive macro-selection, the function of microevolution is mainly to preserve these systems through biological mechanisms, such as copying or amplifying individuals, where stability is key. Many factors, including selection, as well as physical and developmental constraints, are responsible for such stability. Without it, there is no biological evolution. Therefore, by and large, the evolutionary process is about the conservation of information and its carriers, the organisms, unlike the traditional assumption that evolution is mainly about evolving. With increased complexity in the Earth’s history, more layers of regulation will be required. This is why the spiral can gain more layers with increased complexity and biofunctions. For cancer to be successful within stable organisms, a high level of CIN is constantly needed to break the multiple system constraints. Under stress, more factors can contribute to cancer. Increased reports support this idea [47,141].
9. **Clinical Implications:** Karyotype-defined genome architecture has significant implications for disease diagnosis, treatment, and prevention. Strategies targeting genomic stability and the overall karyotype may be more effective than focusing solely on individual gene mutations. For both cancer research and medical use, prioritizing treatment options based on scales of genomics and phases of evolution is crucial [33,34,35,84,142,143,144,145].

**Genome Chaos and Chaotic Evolutionary Process:** Genome chaos represents an information creation and preservation process responsible for new genome system formation. This process involves breaking genome constraints that are ensured by the karyotype coding through the rapid reorganization of genomes (macroevolution), followed by preserving the selected genome through population growth (microevolution). This process can be observed as the transition between disorder and order, combining both certainty and uncertainty, serving as a biological example of chaos. Genome chaos includes many “new” karyotypic alterations, such as “chromothripsis”, “chromoplexy”, “chromosome catastrophes”, “structural mutations”, “chromoanagenesis”, “chromoanasynthesis”, “chromosome fragmentation”, “micronuclei clusters”, and giant nuclei from PGCCs [14,36,72,82,116,142,146,147,148,149,150,151,152,153,154,155,156].

The chaotic evolutionary process is further defined by the certainty and uncertainty of the two-phased evolution model. On one hand, the overall pattern of genome chaos is certain: under crisis conditions, genome reorganization through macroevolution leads to the emergence of new genomes, and their growth leads to dominant cell populations through microevolution. On the other hand, when discussing the triggers of a crisis, the specific mechanisms of genome reorganization, the winners of macroevolution, and which genes will be stochastically used for microevolution, all remain highly uncertain. Like any other chaotic process, the chaotic evolutionary process at the genome level is highly sensitive to initial and ongoing conditions [32,42,75,84,134,135].

**Information Management:** The concept of information management in the context of genomics and evolution consists of four key components:

1. **Information Creation:** This mainly refers to system-level information creation through genome-chaos-mediated karyotype reorganization.
2. **Information Maintenance or Preservation:** Karyotype information is primarily preserved through the functions of sex, the developmental process, and genome integrity.
3. **Information Modification and Transmission:** This involves the accurate replication and distribution of genetic information during cell division and reproduction.
4. **Information Utilization:** This encompasses the processes by which genetic information is accessed and used to produce functional molecules and carry out cellular processes.

By integrating these four components, the concept of information management provides a holistic view of how genomic information is managed to ensure stability, adaptability, information flow, and evolutionary potential. It emphasizes the importance of both the structural (karyotype) and functional (gene expression) aspects of the genome, as well as the dynamic processes that generate and maintain genomic diversity [31,38].

**Organic Codes:** Organic codes refer to the information systems in bio-organisms that encode and regulate various biological processes through making choices. These codes move beyond the genetic code and include other types of biological code that contribute to the organization and functioning of living organisms when information management is involved. The best example is genetic coding, which consists of sequences of nucleotides in DNA that are translated into proteins, with the help of RNA, performing various functions in the cell. Recently, karyotype coding has been proposed for the system level of coding, which organizes the functions of genetically coded genes. The concept of karyotype coding emphasizes the importance of the karyotype—the complete set of chromosomes in a cell—as a fundamental unit of genetic information and a key driver of evolutionary change [30,31,48,49,50,51,157]. Furthermore, it provides a comprehensive framework for understanding the complexity of the genome and its role in disease and evolution. It highlights the importance of considering large-scale chromosomal changes and the overall genomic system, rather than focusing solely on individual gene mutations.

**Supersystem of Cancer:** Cancer populations are often composed of different cellular systems characterized by different karyotypes. These populations are much more complex than those of a given natural species, which share the core genome or karyotype of one species. Cancer cell populations are often mixtures of different cells representing various cellular species, encompassing both macro and microevolutionary phases, with a high level of inter- and intraspecies heterogeneity. We refer to them as a supersystem [107], which is highly dynamic and robust in terms of change and emergence, especially under medical treatment [32].

**Spiral of Life Function Model:** The spiral model illustrates the historical continuity between macroevolution, microevolution, evolutionary innovations, and increased complexity. This model can be used to describe the history of organismal evolution (how major complexities become biological routines one after another), the dynamics of cancer populations (how different new cellular species arise and become dominant through differential growth), and various mechanisms within a given species (how species form by reorganizing system coding and growing populations via microevolution).

In the spiral of life function model, the order of appearance of major evolutionary events is represented by rapid color changes and a gradual increase in the size of the spiral. The more complicated the organisms are, the more mechanisms they possess (reflected by colors). The longer history, the larger the population, and the more spirals there are. The initial spiral represents the mechanisms dominant in primary organisms, while the most recent spiral represents the mechanisms observed in the most advanced organisms, with greater functionality and system complexity [32,47]. Even though the spiral model should encompass many layers beyond the three basic ones discussed in this article—including developmental processes, animal body plans, and cognition-based evolution [26,47,158]—for simplicity, we focus only on the three layers most relevant to cancer evolution. More detailed discussions can be found in the 2nd edition of the book *Genome Chaos* [32].
